# Supplementary material for: South African Lagerstätte reveals middle Permian Gondwanan lakeshore ecosystem in exquisite detail
Source: Commun Biol. 2022 Oct 30;5:1154. doi: 10.1038/s42003-022-04132-y (PMC9618562; doi:10.1038/s42003-022-04132-y)
Supplement: Supplementary file 2 — Supplementary Materials [file 42003_2022_4132_MOESM2_ESM.pdf]

# Supplementary Information

## South African Lagerstätte from the middle Permian reveals exquisite Gondwanan lakeshore ecosystem

Rosemary Prevec,* André Nel, Michael O. Day, Robert A. Muir, Aviwe Matiwane, Abigail P. Kirkaldy, Sydney Moyo, Arnold Staniczek, Bárbara Cariglino, Zolile Maseko, Nokuthula Kom, Bruce S. Rubidge, Romain Garrouste, Alexandra Holland, Helen M. Barber-James
*Corresponding author. Email: r.prevec@am.org.za

**Including:**

Supplementary Notes 1, 2
Supplementary Figures 1, 2
Supplementary Tables 1, 2
Supplementary Data 1
Supplementary References

## Supplementary Note 1

### Terrestrial middle Permian localities in South Africa

Two South African localities were previously assigned to the middle Permian: Lawley in Gauteng Province and Cedara in KwaZulu-Natal Province [1]. Lawley is a spectacularly rich fossil site, but it is a geological outlier, and overlies Archaean basement rock. Prior age estimations were based on poorly substantiated biostratigraphic inferences [1]. Similarly, the assignment of the Cedara locality to the middle Permian is equivocal and was based primarily on the presence of elements common to both lower and upper Permian floras elsewhere. The site lies in an area that has been subjected to extensive faulting, has been disrupted by Jurassic intrusives, and is heavily vegetated, making stratigraphic correlations within the KwaZulu-Natal province extremely challenging. We have tentatively included Lawley (2 in Supplementary Figure 1), but have omitted Cedara from our maps, in light of these uncertainties.

In the past, the Hammanskraal deposit (3 in Supplementary Figure 1) was thought to be lower Permian [1–3]. The Hammanskraal Formation was deposited in the Springbok Flats Basin and the stratigraphic context is known from boreholes and coal mines. Recent palynological data suggests that the fossiliferous deposits are either uppermost lower Permian, or lowermost middle Permian [3], and therefore the locality has been tentatively included.

## Supplementary Note 2

### U-Pb data handling and interpretation

All absolute age and uncertainty calculations were performed using Isoplot v. 4.15 [4], and all data are presented in Supplementary Figure 2, Supplementary Table 2 and Supplementary Data 1. The presence of Proterozoic along with syn-depositional Permian zircon grains in the clay layer suggest reworking of volcanic ash, a common phenomenon during the deposition of the lower Karoo Supergroup [5]. Proterozoic and discordant dates are not considered in any further interpretations. There is significant spread among the Permian dates acquired, which makes interpretation of the direct depositional age of the clay layer impossible, whether of tuffaceous origin or not. However, the youngest three coherent $U^{238}$-$Pb^{206}$ dates that overlap at 2σ error, a robust measure of the maximum depositional age [6], have a weighted mean of 268.0 ± 3.1 Ma, which we interpret as the maximum depositional age of the clay layer (Supplementary Figure 2b). Additionally, the TuffZirc algorithm [4] calculates the most coherent age from the Permian population of dates at 272.0 +2.8/-3.3 Ma, which is within 2σ error of the weighted mean, and supports this interpretation. Three individual dates that are younger than 265 Ma do not form coherent clusters and are interpreted as outliers that may have experienced subtle Pb-loss imperceptible at the precision available from LA-ICP-MS [7]. Permian dates that are older than the maximum depositional age likely represent either re-sedimentation of previously deposited volcanic ash or inheritance due to long magmatic zircon residence times [8], or a combination of these phenomena. Since the fossiliferous horizon overlies and is therefore younger than the clay layer, it was deposited sometime after 268.0 ± 3.1 Ma. Similarly, it must be older than overlying 268.5 ± 3.5 Ma to 264.6 ± 1.9 Ma ashes in the Abrahamskraal Formation [9]. Since both upper and lower age constraints for the fossiliferous horizon are indistinguishable at 2σ error, its depositional age is bracketed to ~268 to ~265 Ma pending higher-precision radiometric ages both at the fossil site and for the Waterford and Abrahamskraal formations in this part of the Karoo Basin. This is compatible with the overall stratigraphic framework for these units and the correlation of precisely dated ash layers from elsewhere in the Karoo Basin (Fig. 4).

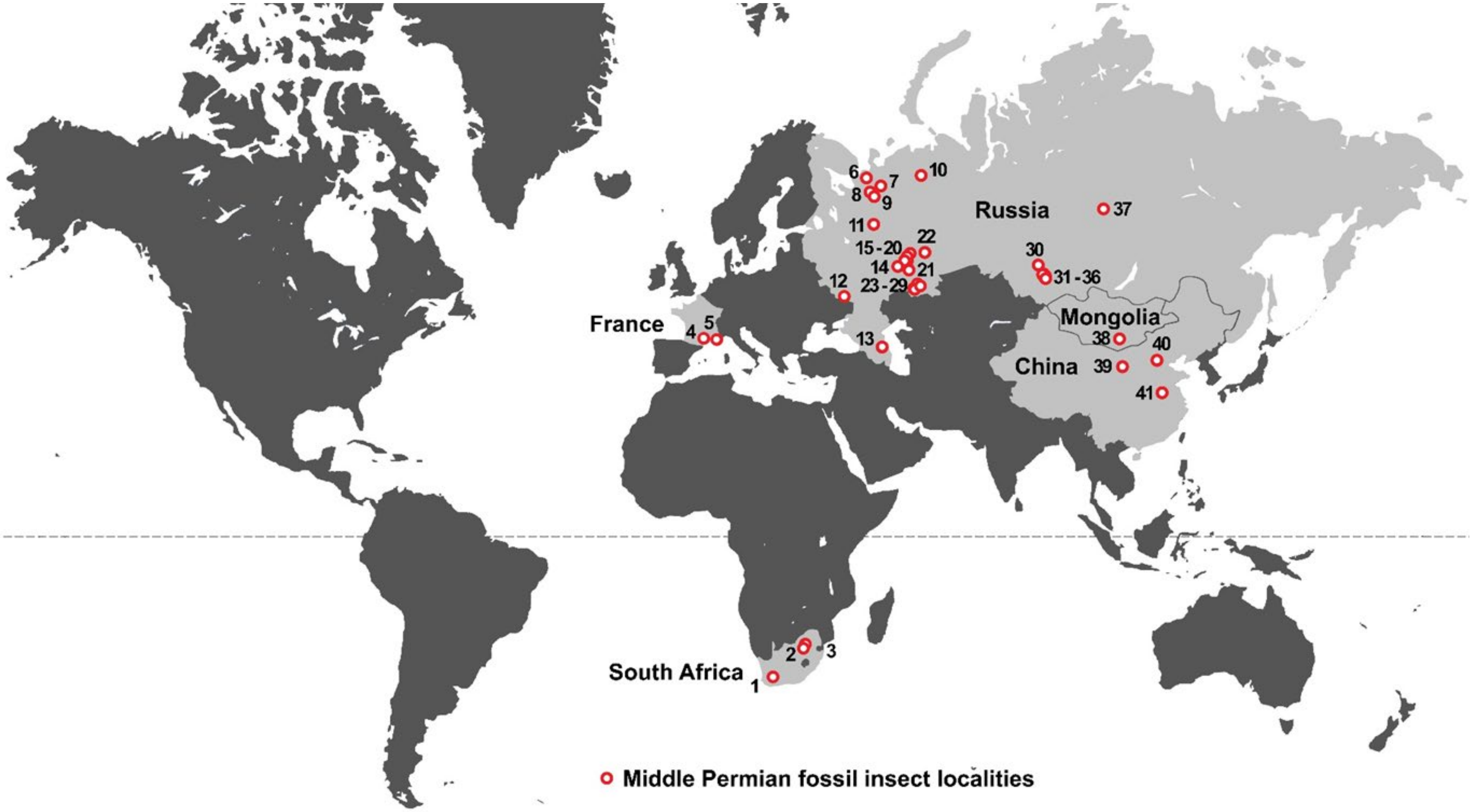


## Supplementary Figure 1.

**Modern map of Earth, with the current positions of middle Permian insect localities indicated.** See Supplementary Table 1 for locality names and references sourced.

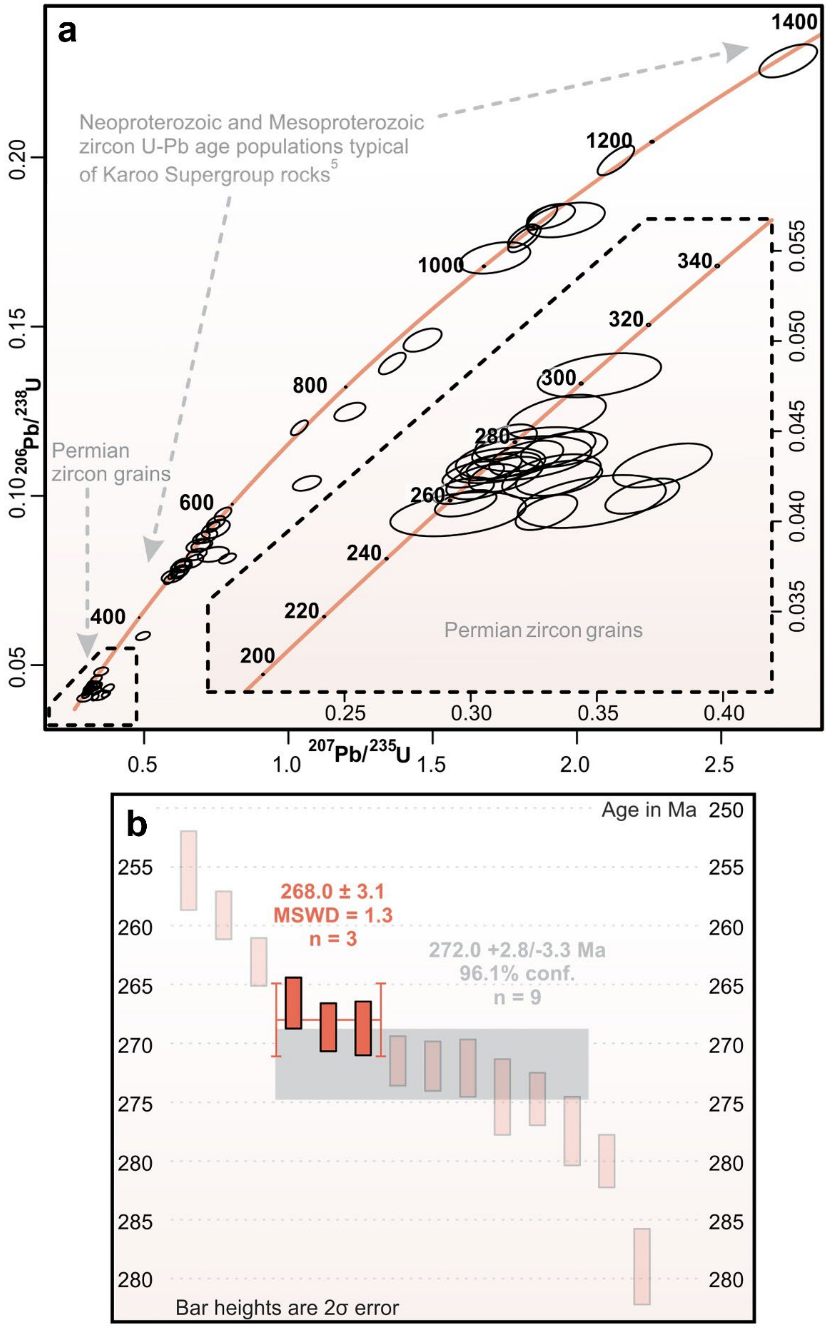


**Supplementary Figure 2. U-Pb zircon geochronology of clay layer that underlies fossiliferous deposits. (a)** All LA-ICP-MS zircon U-Pb radioisotopic data (Data S1) presented on a Wetherill Concordia diagram, with dates ranging from Permian to Mesoproterozoic. Inset shows a close-up of Permian dates only. Data not corrected for common Pb. **(b)** Individual concordant $Pb^{206}/U^{238}$ dates as vertical bars arranged by age. Transparent vertical bars are dates excluded from weighted mean calculation, 268.0 ± 3.1 Ma (MSWD = 1.3), interpreted as maximum depositional age of clay layer. Horizontal grey bar represents TuffZirc age determined by Isoplot

## Supplementary Table 1.

**Details of middle Permian insect localities illustrated in Figure 1 of main text.**

| Map ID # | Stage/Age | Country | Region | Locality name | Geological Formation | Latitude | Longitude | Climatic Zone | References |
|---|---|---|---|---|---|---|---|---|---|
| **1** | Mid-Guadalupian, Wordian; ~268 Ma | South Africa | Northern Cape | Onder Karoo | Abrahamskraal Formation | - 32,410 | 20,670 | cool-temperate | Current publication [10,11] |
| **2** | Geological outlier, poorly understood | South Africa | Gauteng | Lawley | ?Vryheid | - 26.366 | 27.825 | cool temperate | [1,12] |
| **3** | uppermost Kungurian or lowermost Roadian, based on palynological data (273-269 Ma) | South Africa | Gauteng | Hammanskraal | Hammanskraal | - 25,446 | 28,300 | cool temperate | [1]; for age of locality see [3] |
| **4** | Guadalupian | France | Lodève | Lodève, Hérault | Salagou | 43,734 | 3,318 | tropical summer-wet | [13] |
| **5** | Guadalupian | France | Var | Petit Coulet Redon Hill | Gonfaron | 43,510 | 6,630 | tropical summer-wet | [14] |
| **6** | Lower Kazanian; Guadalupian | Russia | Arkhangelsk | Soyana | Iva-Gora beds | 65,776 | 43,396 | sub-tropical desert | [15] |
| **7** | Severodvinian | Russia | Arkhangelsk | Beloshchel'e | Beloshchel'e | 64.915 | 46,980 | sub-tropical desert | [16] |
| **8** | Guadalupian | Russia | Arkhangelsk | Letopala River | Iva-Gora | 63,796 | 45,270 | sub-tropical desert | [17] |
| **9** | Guadalupian | Russia | Arkhangelsk | Sheimo-Gora | Iva-Gora | 64,286 | 44,249 | sub-tropical desert | [18] |

| | | | | | | | | | | |
|---|---|---|---|---|---|---|---|---|---|---|
| **10** | Guadalupian | Russia | North Ural, Komi Republic | Vostochno-Novikbozhskay borehole | Vorkuta Basin, depth int. 1254.3-1261.9 m | 66,020 | 56,890 | warm temperate winter-wet | | 19 |
| **11** | Lower Severodvinian | Russia | Vologda | Kopylovo | Sukhona Formation | 60,352 | 45,065 | sub-tropical desert | | 16 |
| **12** | Guadalupian | Russia | Bolgorod | Bogatyi | Baitugan | 50,658 | 37,880 | tropical summer-wet | | 20 |
| **13** | Guadalupian | Russia | Dagestan | Schuni | ? | 42,194 | 47,172 | tropical summer-wet | | 21 |
| **14** | Guadalupian | Russia | Tatarstan | Sardyk River | Kazan | 55,118 | 51,029 | sub-tropical desert | | 22 |
| **15** | Guadalupian | Russia | Udmurt | Takhtachour ravine | ? | 56,010 | 52,700 | sub-tropical desert | | 22 |
| **16** | Guadalupian | Russia | Udmurt | Tikhiye Gory | Baitugan | 55.892 | 53.338 | sub-tropical desert | | 23 |
| **17** | Wordian (Urzhumian) | Russia | Udmurt | Sarapul'skiy Rayon | ? | 56,407 | 53,386 | sub-tropical desert | | 16 |
| **18** | Urzhumian | Russia | Udmurt Zavyalovski District | Chepanikha | Urzhumian | 56,682 | 53,621 | sub-tropical desert | | 16 |
| **19** | Wordian (Urzhumian) | Russia | Udmurt | Kostovaty | Urzhumian | 56,904 | 54,066 | sub-tropical desert | | 16 |
| **20** | Guadalupian | Russia | Kemerovo | Galevo | Urzhumian | 56,957 | 54,102 | sub-tropical desert | | 24 |
| **21** | Guadalupian | Russia | Bashkortostan | Chatmak Tomak village | Baitugan Formation | 54,660 | 53,800 | sub-tropical desert | | 20 |
| **22** | Guadalupian | Russia | Perm Krai | Tshekarda | Koshelevka | 57,019 | 57,741 | sub-tropical desert | | 25 |
| **23** | Guadalupian Urzhumian | Russia | Orenburg | Kargala | Amanak | 51.866 | 54.839 | sub-tropical desert | | 16,21 |

| | | | | | | | | | | |
|---|---|---|---|---|---|---|---|---|---|---|
| **24** | Guadalupian (early Upper Severodvinian) | Russia | Orenburg | Sakmara | Vyazovka | 51,980 | 55,341 | sub-tropical desert | | 16 |
| **25** | Guadalupian | Russia | Orenburg | Ouralesky Mine | ? | 51,776 | 55,099 | sub-tropical desert | | 26 |
| **26** | Guadalupian (early Upper Severodvinian) | Russia | Orenburg | Cherepanovka | Vyazovka | 52,094 | 56,374 | sub-tropical desert | | 16 |
| **27** | Severodvinian | Russia | Republic of Bashkortostan | Yamansarovo | ? | 52,581 | 55,999 | sub-tropical desert | | 16 |
| **28** | Severodvinian | Russia | Republic of Bashkortostan | Tuembetovo | Vyazovka | 52,343 | 56,484 | sub-tropical desert | | 16 |
| **29** | Guadalupian (early Upper Severodvinian) | Russia | Orenburg | Staroseika | Vyazovka | 52,049 | 56,537 | sub-tropical desert | | 16 |
| **30** | Guadalupian | Russia | Kemerovo | Terekhino | Uskatskaya | 55,332 | 85.555 | cool temperate | | 27 |
| **31** | Guadalupian | Russia | Kemerovo | Uskatskiy well 128 | Roadian | 54,099 | 86,824 | cool temperate | | 28 |
| **32** | Guadalupian | Russia | Kemerovo | Prokopevsk | Kuznetsk | 53,874 | 86,646 | cool temperate | | 29 |
| **33** | Guadalupian | Russia | Orenburg | Chistaya Griva | Il'inskaya Group | 53,770 | 87,140 | cool temperate | | 30 |
| **34** | Guadalupian | Russia | Kemerovo | Abashevo | Mitina | 53.809 | 87.372 | cool temperate | | 30 |
| **35** | Guadalupian | Russia | Kemerovo | Kaltan | Kuznetsk | 53.509 | 87,282 | cool temperate | | 30 |
| **36** | Guadalupian | Russia | Kemerovo | Zelenyi Lug | ? | 53,478 | 87.302 | cool temperate | | 30 |
| **37** | Guadalupian | Russia | Evenkiyskiy AOK, Krasnoyarsk Krai | Kerbo | Degali | 62,436 | 101,600 | cold temperate | | 29 |

| | | | | | | | | | | |
|---|---|---|---|---|---|---|---|---|---|---|
| **38** | Urzhumien; Guadalupian | Mongolia | Ömnögovi Province | Bot-Tologoi /Tavan Tolgoi coal deposit | Tsankhin | 43.625 | 105.474 | cool temperate | | 31 |
| **39** | Guadalupian | China | Hui Autonomous region | Ningxia | Longtan | 106,232 | 106,232 | tropical summer-wet | | 32 |
| **40** | Guadalupian | China | Shanxi province | Yu County | Shihhotse | 38,085 | 113,412 | tropical summer-wet | | 32 |
| **41** | Guadalupian | China | Anhui Province | Houdong: unsure of locality | Yinping | 33.278 | 116,058 | tropical summer-wet | | 32 |

## Supplementary Table 2.

### U-Pb dating method, School of Geosciences, University of the Witwatersrand, South Africa – Analytical set up

| | |
|---|---|
| **Laboratory & Sample Preparation** | |
| Laboratory name | Earthlab, School of Geosciences, University of the Witwatersrand |
| Sample type / mineral | Detrital / Zircons |
| Sample preparation | Conventional mineral separation, 1 inch resin mount, 1 µm polish to finish |
| Imaging | CL, ZEISS Merlin SEM, 11 nA, 10kV, 10 mm working distance at the Central Analytical Facilities, Stellenbosch University |
| **Laser ablation system** | |
| Make, Model & type | AS RESOlution SE Excimer laser |
| Ablation cell & volume | Laurin Technic S-155 dual-volume cell |
| Laser wavelength | 193 nm |
| Pulse width | 5–7 ns |
| Fluence | 2.5 $J/cm^{-2}$ |
| Repetition rate | 7 Hz |
| Spot size | 24 µm |
| Sampling mode / pattern | Static spot ablation |
| Carrier gas | mixed He-Ar atmosphere in the cell, with addition of $N_2$ (5.5 ml/min) after the cell |
| Pre-ablation surface cleaning | 2 pulses, same spot size as during ablation |
| Ablation duration | 29 s |
| Wash-out delay | 7 s |
| Cell carrier gas flow | 0.35 l/min He |
| **ICP-MS Instrument** | |
| Make, Model & type | Thermo Scientific Element XR ICP-MS |
| Sample introduction | Ablation aerosol via conventional tubing |
| RF power | 1350 W |
| Make-up gas flow | 1.0 l/min Ar |
| Detection system | Combination of a single Faraday collector with a SEM (Secondary Electron Multiplier) |
| Masses measured | 202, 204, 206, 207, 208, 232, 238. All masses measured in pulse counting mode, except for $^{238}U$ (analog mode). |
| Integration time per peak (segment duration) | 7, 14, 15, 18, 8, 8, 13 ms, respectively |
| Total integration time per output datapoint | 0.1 s |
| Sensitivity | 0.3% U |
| Dead time | 25 ns |
| **Data Processing** | |
| Gas blank | 12 s on peak prior to each measurement |
| Calibration strategy | GJ-1 used as primary reference material, Plešovice and 91500 used as secondary reference materials |
| Reference Material info | GJ-1 (Jackson et al. 2004; revised isotopic ratios from Horstwood et al. 2016); Plešovice (Slama et al. 2008); 91500 (Wiedenbeck et al. 1995) |
| Data processing package used / Correction for LIEF | Iolite reduction software package v.3.5 with VizualAge; LIEF modelled within each analytical session on the basis of combined analyses of the main reference material; LIEF correction assumes reference material and |

| | |
|---|---|
| | samples behave identically. All ages and uncertainties calculated with Isoplot v. 4.15 (Ludwig 2012) |
| Mass discrimination | Reference material-sample bracketing with $^{207}Pb/^{206}Pb$ and $^{206}Pb/^{238}U$ normalized to zircon GJ-1 |
| Common-Pb correction | No common Pb correction applied to the data |
| Uncertainty level & propagation | Decay constant uncertainties, ratio uncertainty of primary reference material and long-term excess variance of secondary reference material are propagated by quadratic addition. Age uncertainties are quoted at the 2s absolute level. |
| Quality control / Validation | Plešovice: Wtd avg $^{206}Pb/^{238}U$ age = 340.2 ± 0.9 Ma (95% conf, MSWD = 1.4; n = 12); 91500: Wtd avg $^{206}Pb/^{238}U$ age = 1066.6 ± 3.6 Ma (95% conf, MSWD = 0.8; n = 8). |
| | |

## **Supplementary Data 1** (Separate Microsoft Excel file)

U-Pb LA-ICP-MS data from analysis of detrital zircons from a clay layer at base of Onder Karoo fossiliferous bed.

## Supplementary References


1. Anderson, J. M. & Anderson, H. M. *Palaeoflora of Southern Africa. Prodomus of South African megafloras. Devonian to Lower Cretaceous.* (Balkema, 1985).
2. Prevec, R. Elatra: A glossopterid fructification with a bipartite, hooded wing from the lower Permian of Madagascar and South Africa. *Rev. Palaeobot. Palynol.* **210**, 119–139 (2014).
3. Barbolini, N. *et al.* Palynostratigraphic correlation of the Springbok Flats coalfield to other coal-bearing successions in the Karoo basins of southern Africa. *South Afr. J. Geol.* **122**, 1–16 (2019).
4. Ludwig, K. R. Isoplot Version 3.75–4.15: a Geochronological Toolkit for Microsoft Excel. *Berkeley Geochronological Cent. Spec. Publ.* **5**, (2012).
5. Andersen, T., Kristoffersen, M. & Elburg, M. A. How far can we trust provenance and crustal evolution information from detrital zircons? A South African case study. *Gondwana Res.* **34**, 129–148 (2016).
6. Coutts, D., Matthews, W. & Hubbard, S. Assessment of widely used methods to derive depositional ages from detrital zircon populations. *Geosci. Front.* **10**, 1421–1435 (2019).

7. Mundil, R., Ludwig, K. R., Metcalfe, I. & Renne, P. R. Age and Timing of the Permian Mass Extinctions: U/Pb Dating of Closed-System Zircons. *Science* **305**, 1760–1763 (2004).

8. Reid, M. R., Coath, C. D., Harrison, T. M. & McKeegan, K. D. Prolonged residence times for the youngest rhyolites associated with Long Valley Caldera: 230Th-238U ion microprobe dating of young zircons. *Earth Planet. Sci. Lett.* **150**, 27–39 (1997).

9. Lanci, L., Tohver, E., Wilson, A. & Flint, S. Upper Permian magnetic stratigraphy of the lower Beaufort Group, Karoo Basin. *Earth Planet. Sci. Lett.* **375**, 123–134 (2013).

10. Nel, A., Garrouste, R. & Prevec, R. The first Permian Gondwanan damselfly-like Protozygoptera (Insecta, Odonatoptera). *Hist. Biol.* 1–5 (2022) doi:10.1080/08912963.2022.2067996.

11. Cawood, R. *et al.* The first 'Grylloblattida' of the family Liomopteridae from the Middle Permian in the Onder Karoo, South Africa (Insecta: Polyneoptera). *Comptes Rendus Palevol* 451–461 (2022) doi:10.5852/cr-palevol2022v21a22.

12. Rayner, R. J. & Coventry, M. K. A *Glossopteris* flora from the Permian of South Africa. *South Afr. J. Sci.* **81**, 21–32 (1985).

13. Prokop, J. & Nel, A. New Middle Permian palaeopteran insects from Lodève Basin in southern France (Ephemeroptera, Diaphanopterodea, Megasecoptera). *ZooKeys* 41–55 (2011) doi:10.3897/zookeys.130.1311.

14. Garrouste, R., Nel, A. & Gand, G. New fossil arthropods (Notostraca and Insecta: Syntonopterida) in the Continental Middle Permian of Provence (Bas-Argens Basin, France). *Comptes Rendus Palevol* **8**, 49–57 (2009).

15. Martynov, A. V. Permian fossil insects from the Arkhangelsk region: Part 2. Neuroptera, Megaloptera, and Coleoptera, with the description of two new beetles from the Tikhie Mountain. *Tr. Paleozoologicheskogo Instituta Akad. Nauk SSSR* **2**, 63–96 (1933).

16. Aristov, D. S. *et al.* Fossil insects of the middle and upper Permian of European Russia. *Paleontol. J.* **47**, 641–832 (2013).

17. Martynov, A. V. On a new Permian order of orthopteroid insects, Glosselytrodea. *Izv. Akad. Nauk SSSR Otd. Natematicheskikh Estestv. Nauk* **1938**, 187–206 (1938).

18. Becker-Migdisova, E. E. New Permian Homoptera from European USSR. *Tr. Paleontol. Instituta Akad. Nauk SSSR* **76**, 1–112 (1960).

19. Nel, A. *et al.* Revision of Permo-Carboniferous griffenflies (Insecta: Odonatoptera: Meganisoptera) based upon new species and redescription of selected poorly known taxa from Eurasia. *Palaeontogr. Abt. A* **289**, 89–121 (2009).

20. Zalessky, M. D. O. O novykh nasekomykh iz permskikh basseynov rek Kamy, Vyatki i Beloy [On the new insects from the Permian basins of Kama, Viatka and Belaia rivers]. *Tr. Obshchestva Estestvoispyt. Pri Kazansskom Univ.* **52**, 3–30 (1929).

21. Handlirsch, A. Über einige Insektenreste aus der Permformation Russlands. *Zap. Imperatorskoj Akad. Nauk Mém. Académie Impériale Sci. St.-Petersbourg* **16**, 1–7 (1904).

22. Zalessky, M. D. Sur un nouvel insecte Névroptéroïde du Permien du bassin de Kama. *Bull. Société Géologique Fr.* **28**, 381–385 (1928).

23. Zalessky, G. Ancestors of some groups of the present-day insects. *Nature* **140**, 847–848 (1937).

24. Sukacheva, I. & Vassilenko, D. Caddisflies (Insecta, Trichoptera) from the Upper Mesozoic Chernovskie Kopi Locality (Transbaikalia): Part 1. Imago. *Paleontol. J.* **52**, 535–540 (2018).

25. Rasnitsyn, A. P., Aristov, D. S. & Rasnitsyn, D. A. Dynamics of insect diversity during the Early and Middle Permian. *Paleontol. J.* **49**, 1282–1309 (2015).

26. Zalessky, G. Observations sur un nouvel insecte libelluloïde du Permien du Bassin du fleuve Kama. *Ann. Société Géologique Nord* **56**, 36–41 (1931).

27. Becker-Migdisova, E. E. Order Psocoptera. in *Fundamentals of Paleontology: Arthropoda, Tracheata, Chelicerata* (eds. Rohdendorf, B. B. & Davis, D. R.) vol. 9 (Smithsonian Institution Libraries and National Science Foundation, 1961).

28. Martynova, O. M. Novye nasekomye iz Permskikh i Mezozoiskikh otlozhenii SSSR [New insects from Permian and Mesozoic deposits of the USSR.]. *Mater. K Osn. Paleontol.* **2**, 69–94 (1958).

29. Aristov, D. S. Review of the stratigraphic distribution of Permian grylloblattida (Insecta), with descriptions of new taxa. *Paleontol. J.* **43**, 643–651 (2009).

30. Rohdendorf, B. B., Becker-Migdisova, E. E., Martynova, O. M. & Sharov, A. Paleozojskie nasekomye kuznetskogo bassejna [Palaeozoic insects of the Kuznetsk basin]. *Tr. Paleontol. Instituta Akad. Nauk SSSR* **85**, 1–705 (1961).

31. Rasnitsyn, A. P., Aristov, D. S., Gorochov, A. V., Rowland, J. M. & Sinitshenkova, N. D. Important new insect fossils from Carrizo Arroyo and the Permo-Carboniferous faunal boundary. *Bull. N. M. Mus. Nat. Hist. Sci.* **25**, 215–246 (2004).

32. Lin, Q.-B. & Han, W.-J. A new cockroach from the Upper Shihhotse Formation (Upper Permian) in Yu County, HenanProvince. *Acta Palaeontol. Sin.* **24**, 122 (1985).
