# Supplementary material for: South African Lagerstätte reveals middle Permian Gondwanan lakeshore ecosystem in exquisite detail
Source: Commun Biol. 2022 Oct 30;5:1154. doi: 10.1038/s42003-022-04132-y (PMC9618562; doi:10.1038/s42003-022-04132-y)
Supplement: Supplementary file 3 — Description of Additional Supplementary Files [file 42003_2022_4132_MOESM3_ESM.pdf]

Description of Additional Supplementary Files

File name: Supplementary Data 1
Description: U-Pb LA-ICP-MS data from analysis of detrital zircons from a clay layer at base of Onder Karoo fossiliferous bed.
