# Supplementary material for: South African Lagerstätte reveals middle Permian Gondwanan lakeshore ecosystem in exquisite detail
Source: Commun Biol. 2022 Oct 30;5:1154. doi: 10.1038/s42003-022-04132-y (PMC9618562; doi:10.1038/s42003-022-04132-y)
Supplement: Supplementary file 5 — Reporting Summary [file 42003_2022_4132_MOESM5_ESM.pdf]

nature portfolio

Corresponding author(s): Rosemary Prevec

Last updated by author(s): Oct 07, 2022

Reporting Summary

Nature Portfolio wishes to improve the reproducibility of the work that we publish. This form provides structure for consistency and transparency in reporting. For further information on Nature Portfolio policies, see our Editorial Policies and the Editorial Policy Checklist.

Statistics

For all statistical analyses, confirm that the following items are present in the figure legend, table legend, main text, or Methods section.

n/a	Confirmed	
☐	☒	The exact sample size (*n*) for each experimental group/condition, given as a discrete number and unit of measurement
☐	☒	A statement on whether measurements were taken from distinct samples or whether the same sample was measured repeatedly
☒	☐	The statistical test(s) used AND whether they are one- or two-sided<br>*Only common tests should be described solely by name; describe more complex techniques in the Methods section.*
☒	☐	A description of all covariates tested
☒	☐	A description of any assumptions or corrections, such as tests of normality and adjustment for multiple comparisons
☐	☒	A full description of the statistical parameters including central tendency (e.g. means) or other basic estimates (e.g. regression coefficient) AND variation (e.g. standard deviation) or associated estimates of uncertainty (e.g. confidence intervals)
☒	☐	For null hypothesis testing, the test statistic (e.g. *F*, *t*, *r*) with confidence intervals, effect sizes, degrees of freedom and *P* value noted<br>*Give P values as exact values whenever suitable.*
☒	☐	For Bayesian analysis, information on the choice of priors and Markov chain Monte Carlo settings
☒	☐	For hierarchical and complex designs, identification of the appropriate level for tests and full reporting of outcomes
☒	☐	Estimates of effect sizes (e.g. Cohen's *d*, Pearson's *r*), indicating how they were calculated

Our web collection on statistics for biologists contains articles on many of the points above.

Software and code

Policy information about availability of computer code

Data collection	*Provide a description of all commercial, open source and custom code used to collect the data in this study, specifying the version used OR state that no software was used.*
Data analysis	Isoplot R (Vermeesch, 2018) -- https://www.ucl.ac.uk/~ucfbpve/isoplotr/home/index.html<br>Vermeesch, P. (2018). IsoplotR: A free and open toolbox for geochronology. Geoscience Frontiers, 9(5), 1479-1493.

For manuscripts utilizing custom algorithms or software that are central to the research but not yet described in published literature, software must be made available to editors and reviewers. We strongly encourage code deposition in a community repository (e.g. GitHub). See the Nature Portfolio guidelines for submitting code & software for further information.

Data

Policy information about availability of data

All manuscripts must include a data availability statement. This statement should provide the following information, where applicable:

- Accession codes, unique identifiers, or web links for publicly available datasets
- A description of any restrictions on data availability
- For clinical datasets or third party data, please ensure that the statement adheres to our policy

All specimens, photographs and specimen catalogues are lodged at the Albany Museum (Makhanda, South Africa), a provincial museum administered by the Eastern Cape Department of Sports, Recreation, Arts and Culture (DSRAC). All data are available in the main text or the supplementary materials.

Human research participants

Policy information about studies involving human research participants and Sex and Gender in Research.

Reporting on sex and gender	*Use the terms sex (biological attribute) and gender (shaped by social and cultural circumstances) carefully in order to avoid confusing both terms. Indicate if findings apply to only one sex or gender; describe whether sex and gender were considered in study design whether sex and/or gender was determined based on self-reporting or assigned and methods used. Provide in the source data disaggregated sex and gender data where this information has been collected, and consent has been obtained for sharing of individual-level data; provide overall numbers in this Reporting Summary. Please state if this information has not been collected. Report sex- and gender-based analyses where performed, justify reasons for lack of sex- and gender-based analysis.*
Population characteristics	*Describe the covariate-relevant population characteristics of the human research participants (e.g. age, genotypic information, past and current diagnosis and treatment categories). If you filled out the behavioural & social sciences study design questions and have nothing to add here, write "See above."*
Recruitment	*Describe how participants were recruited. Outline any potential self-selection bias or other biases that may be present and how these are likely to impact results.*
Ethics oversight	*Identify the organization(s) that approved the study protocol.*

Note that full information on the approval of the study protocol must also be provided in the manuscript.

Field-specific reporting

Please select the one below that is the best fit for your research. If you are not sure, read the appropriate sections before making your selection.

☐ Life sciences ☐ Behavioural & social sciences ☒ Ecological, evolutionary & environmental sciences

For a reference copy of the document with all sections, see nature.com/documents/nr-reporting-summary-flat.pdf

Ecological, evolutionary & environmental sciences study design

All studies must disclose on these points even when the disclosure is negative.

Study description	The study is primarily descriptive in nature, reporting a hugely significant find that will impact several fields of palaeontological research. We provide preliminary results demonstrating some of the key examples of important temporal and biogeographical range expansions of both plants and invertebrates, and place this discovery within a global context. Team members interrogated taphonomic, geological, geochronological, biostratigraphic, and taxonomic aspects of the locality and the fossils it is yielding.
Research sample	This was entirely constrained by the extent of the fossiliferous zone at the site, degree of weathering of the outcrop, size of team and time available for fieldwork. We are still finding new taxa each time we visit the site, as well as better preserved and more complete specimens. We will continue to collect for years to come..
Sampling strategy	We employed the strategy of bulk-collecting, where we collected everything that was reasonably well preserved. We excavated in such a manor as to aim for the extraction of the largest complete slabs possible. This strategy reduces sampling bias, and aids large-scale ecological data collection. It also increased the chances of sampling organisms that may not be immediately apparent or identifiable due to novelty, orientation or fragmentation. We carefully excavated the site by hand, examining each surface of the fossils with a hand lens.
Data collection	Data on the fossils is primarily descriptive, with preliminary identifications provided by the relevant team experts.
Timing and spatial scale	The fossils considered in the publication were all collected from a single, very small outcrop, over multiple field seasons.
Data exclusions	We have only presented some of the most spectacular finds from the Onder Karoo locality. There are thousands of specimens from the site, including many new taxa, that have not been figured or considered here. The collections will be the subject of many taxonomic papers to come, and new material is still being collected.
Reproducibility	The fossil locality continues to be productive, the collections are available for viewing and study by researchers and members of the public.
Randomization	Not relevant, although see above regarding bulk collection of fossils to mitigate sampling bias.
Blinding	Not relevant, but see above regarding sample collection.

Did the study involve field work? ☒ Yes ☐ No

Field work, collection and transport

Field conditions	Freezing cold to boiling hot! It made no difference to our excavations of the fossil locality, apart from the comfort of our team. We tried to schedule fieldwork for Autumn and Spring months when temperatures were more moderate.
Location	The exact location of the site is protected information, because of the vulnerability of the road-side locality. We have observed fossil thefts and unauthorised excavations in the past. Precise locality information can be requested on an individual basis. The site is about 30 km south-east of Sutherland in the Northern Cape Province, South Africa.
Access & import/export	Material was collected under a South African Heritage Resources Agency (SAHRA) permit (ID # 2310 to R. Prevec), and remains in South Africa, at the Albany Museum, Makhanda, Eastern Cape. Local police and farmers were consulted and made aware of our activities, and consent was given by the owner of the farm adjacent to the site.
Disturbance	Excavations had virtually no impact on the local environment. A very small volume of rock has been removed, and this was done very slowly, carefully and by hand. The site is an abandoned road quarry, and therefore already represents a previously disturbed area.

Reporting for specific materials, systems and methods

We require information from authors about some types of materials, experimental systems and methods used in many studies. Here, indicate whether each material, system or method listed is relevant to your study. If you are not sure if a list item applies to your research, read the appropriate section before selecting a response.

Materials & experimental systems

n/a	Involved in the study
☒	☐ Antibodies
☒	☐ Eukaryotic cell lines
☐	☒ Palaeontology and archaeology
☒	☐ Animals and other organisms
☒	☐ Clinical data
☒	☐ Dual use research of concern

Methods

n/a	Involved in the study
☒	☐ ChIP-seq
☒	☐ Flow cytometry
☒	☐ MRI-based neuroimaging

Palaeontology and Archaeology

Specimen provenance	Material was collected under a South African Heritage Resources Agency (SAHRA) permit (ID # 2310 to R. Prevec).The exact location of the site is protected information, because of the vulnerability of the road-side locality. We have observed fossil thefts and unauthorised excavations in the past. Precise locality information can be requested on an individual basis. The site is about 30 km south-east of Sutherland in the Northern Cape Province, South Africa.
Specimen deposition	All specimens are housed in the fossil collections of the Department of Earth Sciences, Albany Museum, Makhanda, Eastern Cape Province, South Africa.
Dating methods	Dating of the site described in the study was achieved through correlation with strata of known, published age. Additional U-Pb age constraints were obtained through laser ablation induction coupled plasma mass spectrometry (LA-ICP-MS) of zircons from a clay layer directly below the fossiliferous horizon. These provide a maximum depositional age for the fossil horizon, which is compatible with age constraints provided by correlation. LA-ICP-MS analyses were carried out at the University of the Witwatersrand (Earthlab) through the Central Analytical Facility (Stellenbosch University) in South Africa following standard procedures outlined in the supplementary materials. Applied Spectra (AS) RESOlution 193 nm ArF excimer laser system coupled to a Thermo Scientific Element XR magnetic sector-field ICP-MS was used to obtain the isotopic ration. GJ-1 zircon (Jackson et al., 2004; Horstwood et al., 2016) was used as a primary reference material, and Plešovice (Sláma et al., 2008) and 91500 (Wiedenbeck et al., 1995) zircons were used as secondary reference materials. Data validation against secondary reference materials are provided in the supplementary information. Data reduction was performed using Iolite reduction software package (v.3.5; Paton et al., 2011) with VizualAge (Petrus and Kamber, 2012). Data analysis was performed using Isoplot R (Vermeesch, 2018). All data supplied are in accordance with community-derived standards described by Horstwood et al. (2016).<br><br>Horstwood, M.S.A., Košler, J., Gehrels, G., Jackson, S.E., McLean, N.M., Paton, C., Pearson, N.J., Sircombe, K., Sylvester, P., Vermeesch, P., Bowring, J.F., Condon, D.J., Schoene, B., 2016, Community-Derived Standards for LA-ICP-MS U-(Th-)Pb Geochronology – Uncertainty Propagation, Age Interpretation and Data Reporting, Geostandards and Geoanalytical Research, 40, 3, 301–332.<br><br>Jackson, S.E., Pearson, N.J., Griffin, W.L., Belousova, E.A., 2004, The application of laser ablation-inductively coupled plasma-mass spectrometry to in situ U-Pb zircon geochronology, Chemical Geology, 211, 47–69.<br><br>Paton, C., Hellstrom, J., Paul, B., Woodhead, J., Hergt, J., 2011, Iolite: freeware for the visualisation and processing of mass spectrometric data, J. Anal. At. Spectrom., 26, 2508–2518.<br>Petrus, J.A., Kamber, B.S., 2012, VizualAge: A Novel Approach to Laser Ablation ICP-MS U-Pb Geochronology Data Reduction,

Geostandards and Geoanalytical Research, 36, 247-270.

Sláma, J., Košler, J., Condon, D.J., Crowley, J.L., Gerdes, A., Hanchar, J.M., Horstwood, M.S.A., Morris, G.A., Nasdala, L., Norberg, N.,Schaltegger, U., Schoene, B., Tubrett, M.N., Whitehouse, M.J., 2008, Plešovice zircon – a new natural reference material for U-Pb and Hf isotopic microanalysis, Chemical Geology, 241, 1–35.
Vermeesch, P. (2018). IsoplotR: A free and open toolbox for geochronology. Geoscience Frontiers, 9(5), 1479-1493.

Wiedenbeck, M., Allé, P., Corfu, F., et al. 1995. Three natural zircon standards for U-Th-Pb, Lu-Hf, trace element and REE analyses. Geostandards Newsletter, 19, 1–23.

☐ Tick this box to confirm that the raw and calibrated dates are available in the paper or in Supplementary Information.

Ethics oversight

The fossil excavations were performed according to the protocols recommended by the South African Heritage Resources Authority (SAHRA), and in accordance with the permit issued.

Note that full information on the approval of the study protocol must also be provided in the manuscript.
